# Intercropping *Pteris cretica* and *Spinacia oleracea* L. with peanut enhances arsenic removal and soil remediation

**DOI:** 10.3389/fpls.2025.1580332

**Published:** 2025-04-29

**Authors:** Rakhwe Kama, Farhan Nabi, Maimouna Aidara, Peiyi Huang, Muslim Qadir, Sekouna Diatta, Chongjian Ma, Huashou Li

**Affiliations:** ^1^ College of Natural Resources and Environment, South China Agricultural University, Guangzhou, China; ^2^ Laboratory of Ecology, Faculty of Sciences and Technology, Cheikh Anta University, Dakar, Senegal; ^3^ School of Biology and Agriculture, Shaoguan University, Shaoguan, China

**Keywords:** arsenic, hyperaccumulator plant diversity, *Arachis hypogaea*, intercropping system, contaminated soil

## Abstract

Arsenic (As) exposure through agricultural soil contamination poses significant health risks and threatens food security. This study explored the efficacy of hyperaccumulator plant diversity and intercropping systems in enhancing As removal from contaminated soil while simultaneously reducing As uptake in peanuts (*Arachis hypogaea* L.). Thus, a pot experiment was conducted using As-contaminated soil, peanut plants, and hyperaccumulator species as the experimental materials. The experimental treatments included monocultured peanuts (Ck) and peanuts intercropped with *Pteris cretica*. (P*Pc), intercropped peanut with *Spinacia oleracea* L. (P*So), and intercropped peanut with *P. cretica* and *S. oleracea* L. (P*Pc*So). Our findings revealed that the intercropping system significantly reduced soil As levels compared to monocropping. In addition, peanut As uptake was significantly decreased in hyperaccumulator plants, with enhanced effects under hyperaccumulator plant diversity, minimizing the risk of As transfer to the food chain. Moreover, the As removal rate was higher under intercropping than under monocropping, with the highest removal rate of 88% under intercropped peanut/*P. cretica*/*S. oleracea* L., followed by peanut/*S. oleracea* L. (81%) and peanut/*P. cretica* (80%). The results demonstrate the potential of using diverse hyperaccumulator plants and intercropping systems as sustainable and effective methods for remediating As-contaminated soils, while simultaneously ensuring food safety. However, further research is needed to elucidate the underlying mechanisms driving these effects and to optimize the phytoremediation of As-contaminated soil and crop production.

## 1 Introduction

Soil contamination by heavy metals (HMs) is an escalating environmental concern, owing to the rapid development of industrial mining and agricultural production. This pollution disrupts the natural ecosystem cycle and threatens human and environmental health (Shetty et al., 2023). For instance, HMs, such as lead, mercury, cadmium, and arsenic (As), are particularly hazardous and can damage human health (Balali-Mood et al., 2021; Kama et al., 2023a, b). Therefore, it is essential to find alternatives for HM-contaminated soil remediation.

Several remediation techniques have been adopted based on the status of the contaminated area and the available financial resources (Rajendran et al., 2022; Shen et al., 2022; Lee et al., 2024). For instance, phytoremediation utilizing hyperaccumulator plants has become a well-known concept because of its low cost, effectiveness, and eco-friendly properties (Bhat et al., 2022; Kama et al., 2023c). Hyperaccumulator plants serve as the foundation for phytoextraction and practical applications in polluted soil decontamination (Li et al., 2023; Wang et al., 2023; Fadzil et al., 2024). Hyperaccumulator plants are globally distributed (Reeves et al., 2018). Owing to its high biodiversity, China is considered a worldwide hotspot for hyperaccumulator plants (Li et al., 2018; Kama et al., 2023c). Several hyperaccumulator plant species of various HMs have been discovered in China over the last two decades (Reeves et al., 2018). These hyperaccumulator plants have been successfully used in the rerstoration of contaminated soil because of their high HM accumulation capacity (Rehman et al., 2017; Bhat et al., 2022; Fadzil et al., 2024). For example, the roots of hyperaccumulator plants can reduce soil HM concentration by absorbing and transferring high concentrations to the aerial parts (Rehman et al., 2017). However, soil type and the identity and diversity of cultivated plants can influence hyperaccumulator HM uptake.

HM phytoextraction involves the use of specific plants with a high capacity to absorb HMs from contaminated soil, transfer them, and store them in their aboveground parts, such as stems and leaves (Bahsaine et al., 2022; Kama et al., 2023c). Continuous use of hyperaccumulators can effectively reduce soil HM concentrations to permissible limits (Kama et al., 2023c). This method provides various advantages, such as low cost, upholding soil structure, and the ability to remediate large areas (Adeoye et al., 2022). Consequently, the use of hyperaccumulators, such as *Amaranthus hypochondriacus* L., *Pteris cretica*, and *Spinacia oleracea* L., has gained widespread recognition in recent years (Pavlík et al., 2010; Veladzic et al., 2015). For instance, *A. hypochondriacus* L. has been to be found effective for HM removal, such as cadmium, from contaminated soils (Wang et al., 2019). Additionally, *P. cretica* is characterized by its ability to absorb As and store it in its tissues, with higher concentrations often found in roots (Pavlík et al., 2010; Jeong et al., 2015; Veladzic et al., 2015; Fayiga and Saha, 2016). Therefore, *P. cretica* is a promising plant species for As-contaminated remediation and is considered necessary for environmental and ecological restoration (Eze and Harvey, 2018). Moreover, research has shown that *S. oleracea* L., a nutritious leafy vegetable from central Asia, can absorb HMs such as As and Cd from soil, preventing them from being taken up by other crops (Salaskar et al., 2011; Alka et al., 2021). Previous studies have highlighted that *S. oleracea* L. is a reliable absorber of HM, exhibiting notable accumulation of HMs such as As and mercury (Hg) (Salaskar et al., 2011). Overall, *S. oleracea* L. can be considered a reliable option for HM removal from contaminated soils. Using HM hyperaccumulators such as *P. cretica* and *S. oleracea* L. for HM removal is a sustainable and eco-friendly solution for reducing the negative impacts of HM pollution. However, further studies are needed to investigate the effects of hyperaccumulator identity and diversity on As-contaminated soil in a peanut/hyperaccumulator intercropping system.

Intercropping systems involve concurrent growth of two or more crops (Hong et al., 2017; Fu et al., 2019; Baldé et al., 2020). It is a popular and widely used cultivation technique for maximizing available cultivable land use and other resources. To effectively manage crop combinations under various environmental conditions, a thorough understanding of nutrient distribution mechanisms between intercropped plants is essential. The efficiency of intercropping systems in improving soil health and crop production has recently received substantial attention (Baldé et al., 2020; Kang et al., 2020; Ma et al., 2022; Kama et al., 2023a, b). Intercropping can significantly reduce the uptake of heavy metals in co-cultivated crops through several mechanisms (Kama et al., 2023b; Liu et al., 2023). For instance, when different crops are intercropped, their root systems often occupy different soil layers, reducing competition for heavy metal uptake (Zhang et al., 2024). Additionally, some plant species can immobilize or even hyperaccumulate certain heavy metals, thereby reducing the amount of these metals available for uptake by other plants (Rascio and Navari-Izzo, 2011). Furthermore, it has been found that intercropping can also enhance microbial activity in the rhizosphere, leading to increased heavy metal immobilization through complexation and precipitation reactions (Kama et al., 2023b). Overall, intercropping can be an effective strategy for mitigating the uptake of heavy metals in co-cultivated crops. Intercropping consumable crops with HM hyperaccumulator plants in contaminated soil has become a popular practice (Hong et al., 2017; Li et al., 2019; Baldé et al., 2020; Kang et al., 2020; Kama et al., 2023a). Intercropping hyperaccumulator plants with consumable crops can reduce toxic HM concentrations in the soil without significantly affecting the growth and yield of the co-cultivated crop (Kama et al., 2023c). This cultivation method rejuvenates the ecological and environmental benefits of soil in cultivated areas (Baldé et al., 2020; Pelech et al., 2021; Liu et al., 2022; Ma et al., 2022). Therefore, understanding and assessing the effects of crop/hyperaccumulator plants’ intercropping system on As removal and movement under soil–plant interactions are crucial in facilitating the phytoremediation of As-contaminated soil.

Most phytoremediation studies on As removal have focused on monocultures or single-species intercropping of hyperaccumulators, with limited research on the synergistic effects of plant diversity on intercropping systems. While *P. cretica* and *S. oleracea* L. have been studied for As accumulation, their combined effects in intercropping systems remain unexplored. This study aims to address this gap by investigating the effects of intercropping *P. cretica* and *S. oleracea* L. with *Arachis hypogaea* L. (peanut) on As removal from contaminated soils and peanut As uptake. We hypothesized that intercropping with diverse hyperaccumulator species would enhance As removal from soil and significantly reduce As uptake in peanuts, with the most pronounced effect observed in the system involving both *P. cretica* and *S. oleracea* L. The objectives of this study were to 1) evaluate the effectiveness of intercropping *P. cretica* and *S. oleracea* L. with *A. hypogaea* L. to enhance As removal from contaminated soils; 2) assess the impact of this intercropping system on reducing As uptake in peanuts; and 3) determine whether the diversity of hyperaccumulator species leads to synergistic effects, improving overall As remediation efficiency compared to monocropping systems.

## 2 Materials and methods

### 2.1 Soil collection and characteristics

The experimental soil was collected from the Demonstration Base for Safe Utilization and Restoration of Cultivated Land at the Northern Guangdong Research Station located in Tangxin Village, Xinjiang Town, Shaoguan City, Guangdong Province (24°29°31”N; 113°48°39”E). Shaoguan City is a mining region covering the Dabaoshan mining area, which is about 578.96 hm^2^ and spans Qujiang and Wengyuan Counties. The collected soil was mixed thoroughly, air-dried, and ground to pass through a 2 mm mesh for the pot experiment. The basic chemical properties of the experiment were as follows: pH 6.54, EC: 218 µS cm^−1^, OM: 29.91 g kg^−1^, and total As: 27.86 mg kg^−1^.

### 2.2 Site description and experimental design

This study was conducted in the Natural Resources and Environment College greenhouse at South China Agricultural University. For the experiment, plastic boxes (65 cm × 48.5 cm × 40 cm) were filled with 40 kg of soil mixed with 80 g of compound fertilizer containing at least 29% nutrients, N-P_2_O_5_-KO_2_. Peanut (P), *P. cretica* (PC), and *S. oleracea* L. (SO) were the used plants for the pot experiment. Peanut seeds were collected from the Agronomy Courtyard of South China Agricultural University, while *P. cretica* and *S. oleracea* L. were obtained from the South China Botanic Garden.

The experimental treatments include 1) monocropped peanut (Ck), 2) intercropped peanut with *Pteris cretica* (P ∗ Pc), 3) intercropped peanut with *S. oleracea* L. (P ∗ So), and 4) intercropped peanut with *Pteris cretica* and *Spinacia oleracea* L. (P ∗ Pc ∗ So). Each treatment was replicated five times. Seeds were sown in different seedbeds, and uniform-sized seedlings were transplanted into pots after 10 days of growth. The number of peanut plants in monocropping was six plants per pot, whereas the ratio of peanut:hyperaccumulator plants under the intercropping system was 3:3 and 2:2:2 under one and two hyperaccumulator plant species richness, respectively. Tap water from the station was used for irrigation. Plants were watered every 2 days to achieve a soil water-holding capacity of 70% during cultivation. During the cultivation period, the temperature inside the greenhouse was between 25°C and 34°C, while the relative humidity was between 27% and 48%.

### 2.3 Plant and soil sampling

Peanut plant growth parameters were measured three days before harvest. Leaf chlorophyll content (Lchl) and leaf nitrogen (LN) levels were determined using a hand-held plant nutrient meter. After 8 weeks of growth, the peanut and hyperaccumulator plants were harvested and separated into roots and shoots. All samples were washed with deionized water, dried in an oven at 70°C until they reached a constant weight, and ground for chemical analysis.

Rhizosphere soil was collected by shaking the roots, while bulk soil was collected directly from the plastic boxes. The collected soil samples were air-dried, sieved (2 mm mesh), thoroughly mixed, and then divided into two subsamples: the first subsample was used for As species analysis and the other for chemical analysis.

### 2.4 Plant and soil chemical analysis

To determine As concentration in plants, 0.10 g–0.20 g of plant material was digested using 8 mL of concentrated HNO_3_ and 2 mL of 30% H_2_O_2_ in a microwave digestion system (CEM Mars6, USA). The samples were diluted with deionized water to a volume of 25 mL and filtered after digestion. The As concentration was measured using an atomic fluorescence spectrophotometer (AFS-933, Beijing Jitian, China).

To determine the soil’s basic chemical properties, 10 g of air-dried soil was weighed and mixed with 25 mL of deionized water. The mixture was then shaken for 1 h and allowed to settle for 30 min. The soil pH was measured using a pH meter (Shanghai Sanxin Instrument Factory) (Kama et al., 2023a; Kama et al., 2024). Soil EC, OM, redox potential (Eh), and soil nutrient content were determined following the approaches described by Chen et al. (2023) and Kama et al. (2023b).

Soil As^3+^, As^5+^, and total As were determined by mixing 0.4 M H_3_PO_4_ and 0.4 M NaOH, following the extraction method described by Qin et al. (2023). The As fractions F1 (non-specifically sorbed), F2 (specifically sorbed), F3 (amorphous and poorly crystalline hydrous oxides of Fe and Al), F4 (well-crystallized hydrous oxides of Fe and Al), and F5 (residual phases) were extracted and determined from the soil using the methods described by Chen et al. (2023).

### 2.5 Calculations and statistical analysis

The relative translocation of As from soil to other parts of the plant species was determined by calculating the translocation factor (TF) (Cao et al., 2019).

The relative mobility and accumulation of arsenic were evaluated using several indices, including bioconcentration factor (**Equation 1**), translocation factor (**Equation 2**), removal rate (**Equation 3**), and extraction efficiency (**Equation 4**).


(1) $$\text{Bioconcentration factor }\left(\text{BCF}\right)\;=\frac{\text{Concentration of As in roots}}{\text{ As concentration in soil }}$$

(2) $$\text{Translocation factor }\left(\text{TF}\right)=\frac{\text{Concentration of As in aerial parts}}{\text{Concentration of As in corresponding root }}$$

(3) $$\text{Removal rate }\left(\%\right)=\frac{\text{decrease in soil As content after planting }}{\text{initial total As content in soil }}\times 100$$

(4) $$\text{Extraction efficiency}\;\left(\%\right)=\frac{\text{As extracted by plants}}{\text{initial total As content in soil }}\times 100$$

#### 2.5.1 Determination of optimal planting pattern using TOPSIS

The Technique for Order of Preference by Similarity to Ideal Solution (TOPSIS) (Lai and Hwang, 1994) was used to determine the best treatment based on plant height, removal rate, and As uptake by the peanut plants. The steps required to determine the best planting pattern that balances the detrimental effects of As uptake by peanut plants and the beneficial effects of increasing plant height and As removal rate have been described in our previous article (Kama et al., 2023a).

All statistical analyses were performed using SPSS software (version 19.0, SPSS Inc., Chicago, IL, USA). One-way ANOVA was conducted to compare the effects of hyperaccumulator plant presence and planting pattern on peanut plant growth parameters, As concentration in soil and plants, As BCF, and TF. Furthermore, two-way ANOVA was performed to determine any differences between the hyperaccumulators, planting patterns, and their interactive effects with various treatments on plant growth parameters. Statistical significance was set at p<0.05, unless otherwise stated. Origin 2021b was used for figure production. To examine the correlation between basic soil chemical properties and total As, principal component analysis (PCA) was employed. Finally, a Pearson’s correlation analysis was conducted to evaluate the relationship between the As removal rate, extraction efficiency, and soil basic chemical properties.

## 3 Results

### 3.1 Effects of planting patterns on peanut growth traits

Peanut growth traits were affected by the presence of hyperaccumulator plants and the planting pattern (**Table 1**). A slight decrease in peanut growth parameters was noted under mixed peanut/hyperaccumulator plant cultures compared with single-cropped peanuts. Peanut plant height was higher under monocropping system compared to intercropping treatments. Similarities were observed in shoot dry weight (SDW). In contrast, the presence of hyperaccumulator plants and planting patterns had no significant effect on the peanut stem diameter. Peanut leaf chlorophyll content (Lchl) was also higher under monocropped peanuts followed by intercropped peanuts with *P. cretica*. No significant differences were observed in peanut leaf nitrogen (LN) levels based on planting patterns. These results suggest that peanut growth traits decrease slightly in the presence of hyperaccumulators.

**Table 1:** Peanut growth traits.

Treatments	PH (cm)	STD (mm)	SDW (g)	RDW (g)	R/S	Chl (SPAD)	NL (mg L^-1^)
Ck	23.67 ± 0.58a	1.5 ± 0.01a	20.8 ± 1.09a	1.9 ± 0.01b	0.11 ± 0.001a	21.7 ± 0.43a	2.10 ± 0.21a
P*Pc	21.47 ± 0.24ab	1.5 ± 0.01a	18.2 ± 0.76b	2.1 ± 0.03a	0.12 ± 0.001a	21.3 ± 0.27a	2.04 ± 0.08a
P*So	21.13 ± 0.41ab	1.4 ± 0.01ab	19.3 ± 0.89ab	2.1 ± 0.01a	0.118 ± 0.001a	20.3 ± 0.32b	2.20 ± 0.14a
P*Pc*So	20.50 ± 0.29b	1.2 ± 0.01b	17.9 ± 0.54c	1.9 ± 0.03b	0.10 ± 0.001a	20.3 ± 0.51b	2.11 ± 0.12a
PT	ns	ns	*	ns	ns	*	ns
PP	***	**	**	ns	ns	*	ns
PT*PP	**	*	*	ns	ns	ns	ns

Ck: refers to monocropped peanut, P*Pc refers to intercropped peanut and *Pteris cretica*, peanut grown with (P*So) refers to intercropped peanut and *Spinacia oleracea* L., and P*Pc*So refers to intercropped peanut, *Pteris cretica*, and *Spinacia oleracea* L.; PH, plant height; STD, stem diameter; SDW, shoot dry weight; RDW, root dry weight; R/S, root-shout ratio; Chl, leaf chlorophyll content; LN leaf nitrogen level; PT, plant type; and PP, planting pattern.

Each value represents the mean of five replicates, and the different letters indicate a significant difference at *p*< 0.05 (ns, non-significant, * *p<* 0.05, *** *p<* 0.01, *p<* 0.001).

### 3.2 Effects of planting patterns on soil properties

#### 3.2.1 Effects of planting patterns on soil basic chemical properties

**Figure 1:**
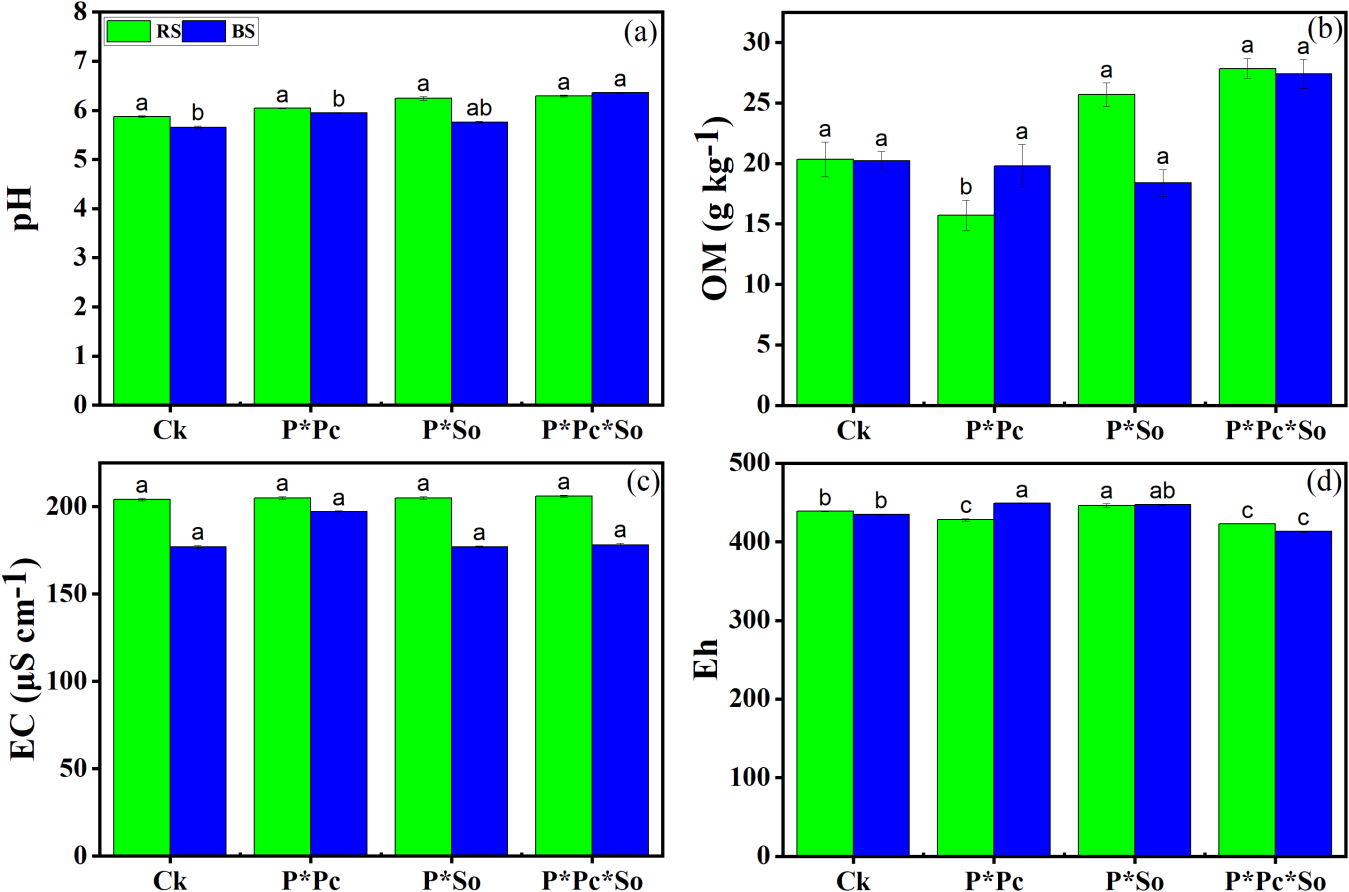
Fundamental chemical properties of the soil, including pH **(a)**, organic matter **(b)**, electric conductivity **(c)**, and cation exchange capacity **(d)**. Data are presented as means ± standard errors (*n* = 5). Lowercase letters in the same column indicate significant treatment differences (*p* <0.05). Ck: refers to monocropped peanut, P ∗ Pc refers to intercropped peanut and *Pteris cretica*, peanut grown with (P ∗ So) refers to intercropped peanut and *Spinacia oleracea* L., and P ∗ Pc ∗ So refers to intercropped peanut, *Pteris cretica*, and *Spinacia oleracea* L.

Changes in the basic chemical parameters of the soil are shown in **Figure 1**. A slight increase in basic soil chemical parameters was noted in the rhizosphere soil compared to the bulk soil (**Figure 1**). Regarding soil pH and organic matter (OM), there was no discernible variation based on planting pattern or type of hyperaccumulator plant (**Figures 1a, b**). Similar trends were observed in soil EC except under peanut–*P. cretica* intercropping system, where a slight increase was observed compared with other treatments (**Figure 1c**). The soil redox potential (Eh) decreased under hyperaccumulator plant diversity compared with the other planting patterns (**Figure 1d**). Overall, this study demonstrated that planting pattern had a slight impact on the soil’s basic chemical properties.

#### 3.2.2 Effects of planting patterns on soil nutrient content

**Figure 2:**
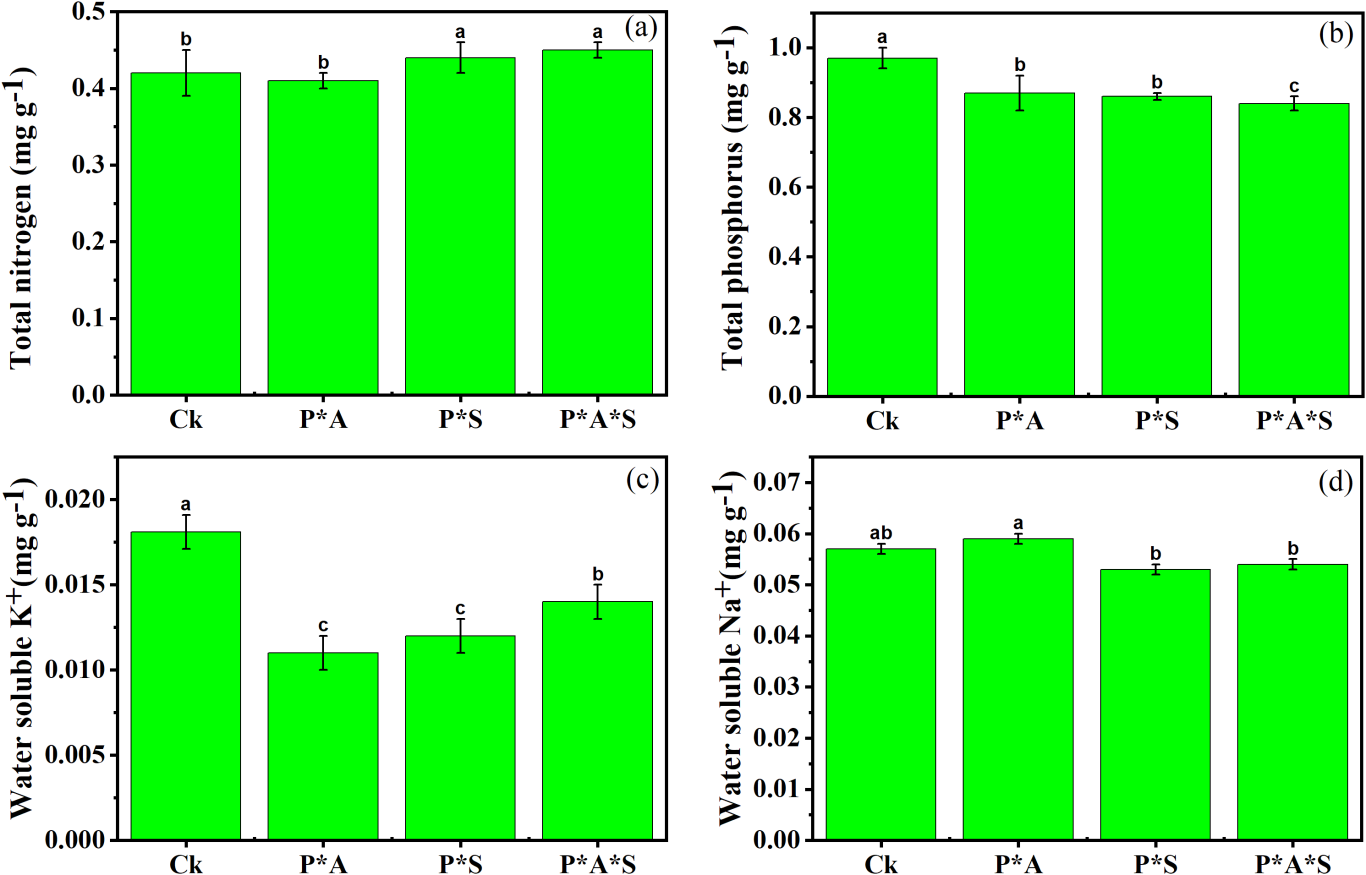
Soil nutrient content, including total nitrogen **(a)**, total phosphorus **(b)**, water-soluble potassium (K^+^) **(c)**, and water-soluble sodium (Na^+^) **(d)**. Data are presented as means ± standard errors (*n* = 5). Lowercase letters in the same column indicate significant treatment differences (*p* <0.05). Ck: refers to monocropped peanut, P ∗ Pc refers to intercropped peanut and *P. cretica*, peanut grown with (P ∗ So) refers to intercropped peanut and *Spinacia oleracea* L., and P ∗ Pc ∗ So refers to intercropped peanut, *Pteris cretica*, and *Spinacia oleracea* L.

The changes in the soil nutrient content across the different treatments are shown in **Figure 2**. The concentration of total nitrogen (TN) in soil was higher under intercropped peanuts with *P. cretica* and *S. oleracea* L. compared to monocropped peanut and peanut intercropped with only one hyperaccumulator plant. The opposite situation was observed regarding soil total phosphorus (TP) concentration, with a significant decrease in the presence of hyperaccumulator plants. The highest concentration of water-soluble K^+^ was recorded in monocropped peanut soil (Ck) compared to other treatments. In contrast, the concentration of water-soluble Na^+^ was higher in the peanut–*P. cretica* intercropping system.

### 3.3 Concentrations of As species and total As in soil

**Figure 3:**
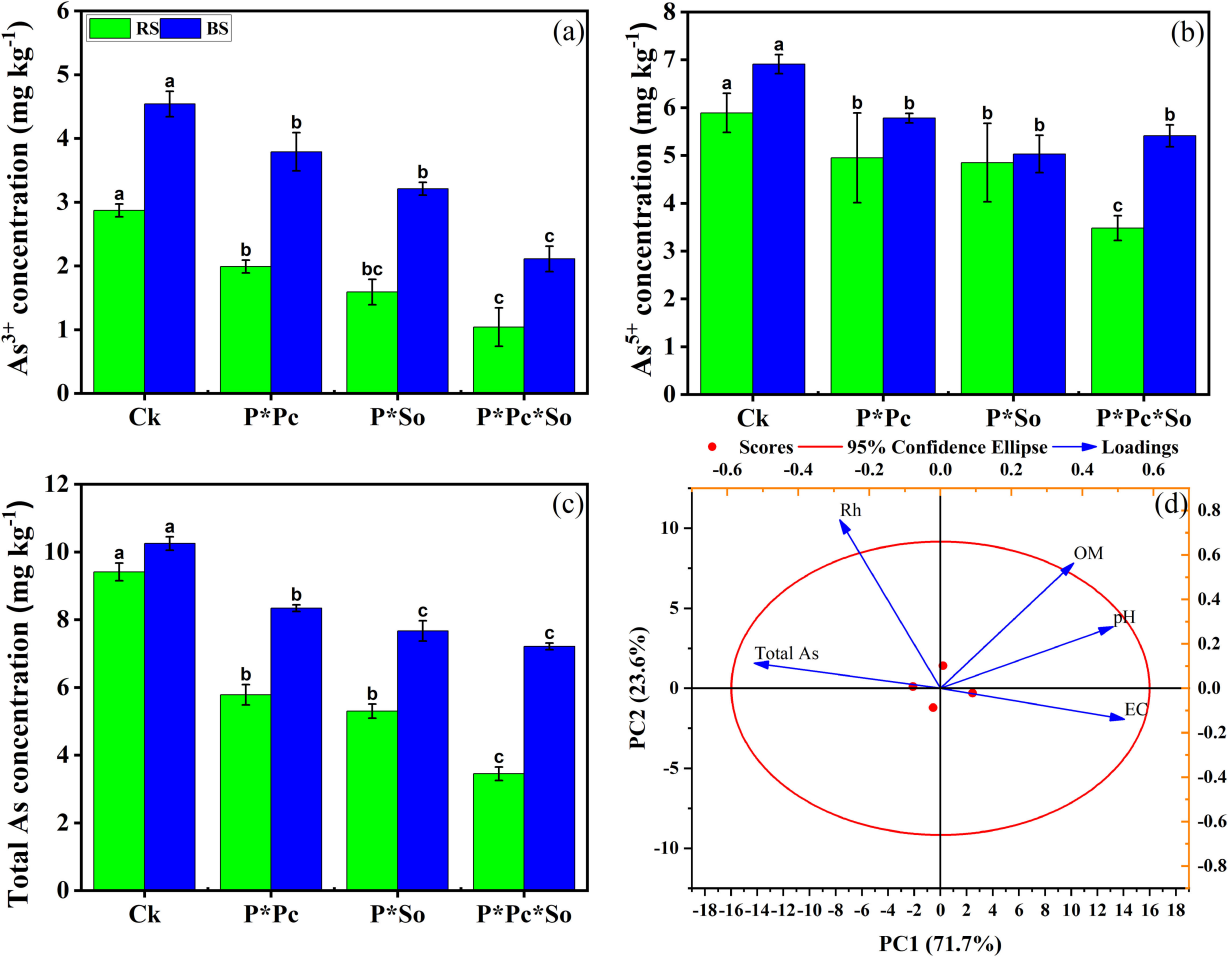
Concentration of As^3+^ **(a)**, As^5+^ **(b)**, and total As in soil **(c)** and Principal component analysis (PCA) of soil basic chemical properties, and total As concentration in soil **(d)**. Data are presented as means ± standard errors (*n* = 5). Lowercase letters in the same column indicate significant treatment differences (*p* <0.05). RS refers to rhizosphere soil, SB refers to bulk soil; Ck: refers to monocropped peanut, P ∗ Pc refers to intercropped peanut and *Pteris cretica*, peanut grown with (P ∗ So) refers to intercropped peanut and *Spinacia oleracea* L., and P ∗ Pc ∗ So refers to intercropped peanut, *Pteris cretica*, and *Spinacia oleracea* L.

**Figure 3** illustrates the variation in As species and total As concentrations across the different treatments. Significant decreases were observed in As species and total As concentrations in the soil based on the planting patterns and the type of cultivated plants. The concentration of total As was reduced in the intercropped peanut/*P. cretica* and/or peanut/*S. oleracea* L. compared with monocropped peanut. In addition, significant effects were observed under hyperaccumulator plant diversity, where the concentration of total As was lower than that in all other treatments (**Figure 3c**).

Notable trends were consistently observed for both the As^5+^ and As^3+^ concentrations, as shown in **Figures 3a, b**. Principal component analysis (PCA)was used to analyze the correlation between the total As and basic soil chemical properties (**Figure 3d**). The first two principal components had a cumulative contribution ratio of 93.8%. PCA showed that the decrease in soil total As was positively correlated with soil basic chemical properties, except for the redox potential. This study shows that the presence of hyperaccumulator plants decreases As concentration in soil, with more pronounced impacts under hyperaccumulator plant diversity.

### 3.4 Changes in As concentration in plant’s various organs, BCF, and TF

**Figure 4:**
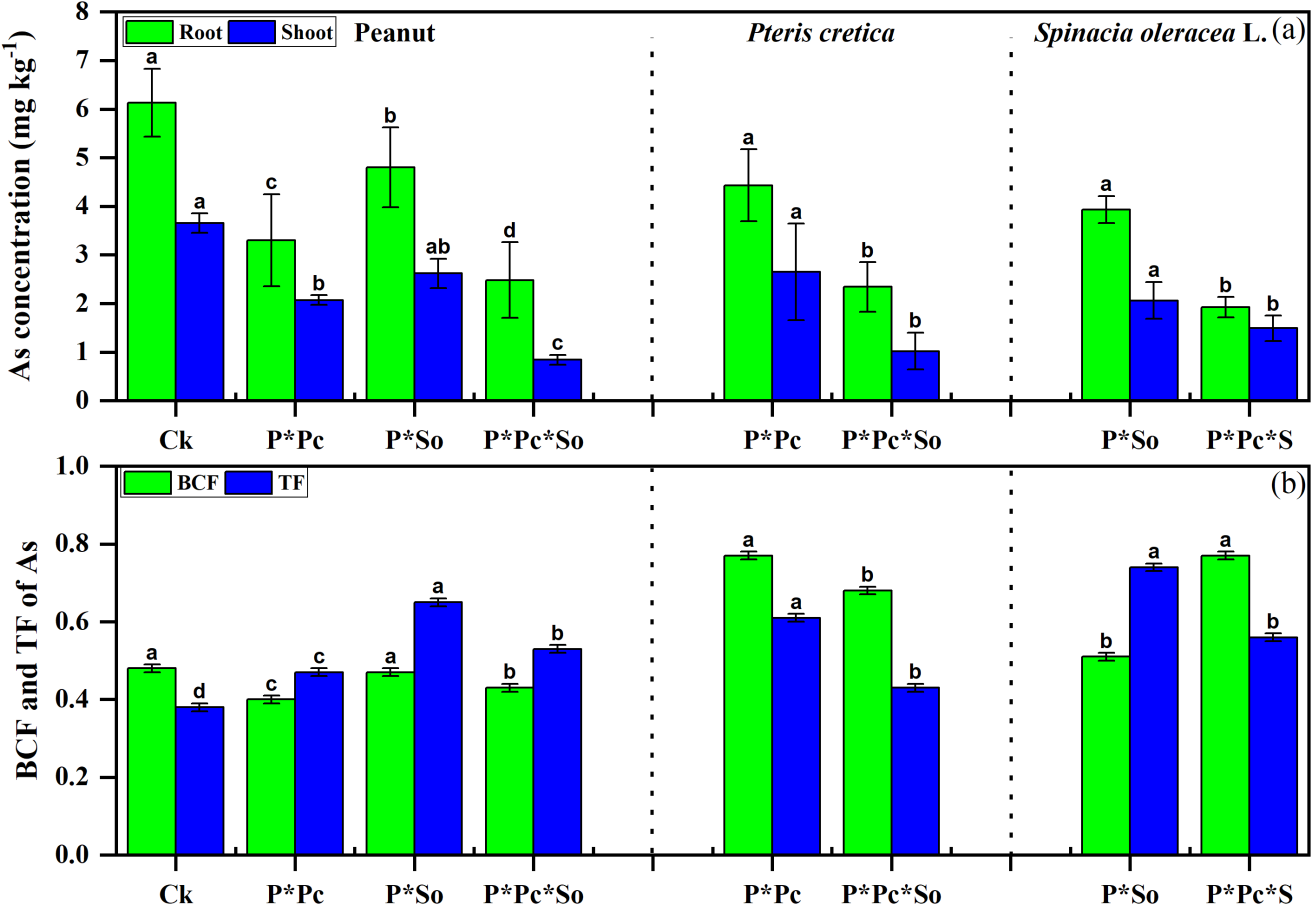
Concentrations of As in peanut and hyperaccumulator plant various parts **(a)** and As mobility in soil plant-system **(b)**. Data are presented as means ± standard errors (*n* = 5). Lowercase letters in the same column indicate significant treatment differences (*p* <0.05). Ck: refers to monocropped peanut, P ∗ Pc refers to intercropped peanut and *Pteris cretica*, peanut grown with (P ∗ So) refers to intercropped peanut and *Spinacia oleracea* L., and P ∗ Pc ∗ So refers to intercropped peanut, *Pteris cretica*, and *Spinacia oleracea* L.

The concentrations of As in various plant organs, as well as in the BCF and TF, are shown in **Figure 4**. Higher concentrations of As and BCF were observed in monocropped peanuts than in other treatments. In addition, As concentrations were higher in roots than in shoots. A decrease in As concentration in various peanut organs was observed in hyperaccumulator plants, with enhanced effects under hyperaccumulator plant diversity. A decreasing trend was observed in the BCF values, in contrast to TF, which increased under the intercropping system. The As concentration in hyperaccumulator plants was also reduced under peanut intercropping with combined hyperaccumulator plants compared to under a single hyperaccumulator/peanut intercropping system. However, a decrease in TF was observed in both hyperaccumulator plants while the BCF increased. This study shows that hyperaccumulator presence and diversity reduce As uptake in peanuts.

### 3.5 Removal rate and extraction efficiency

**Figure 5:**
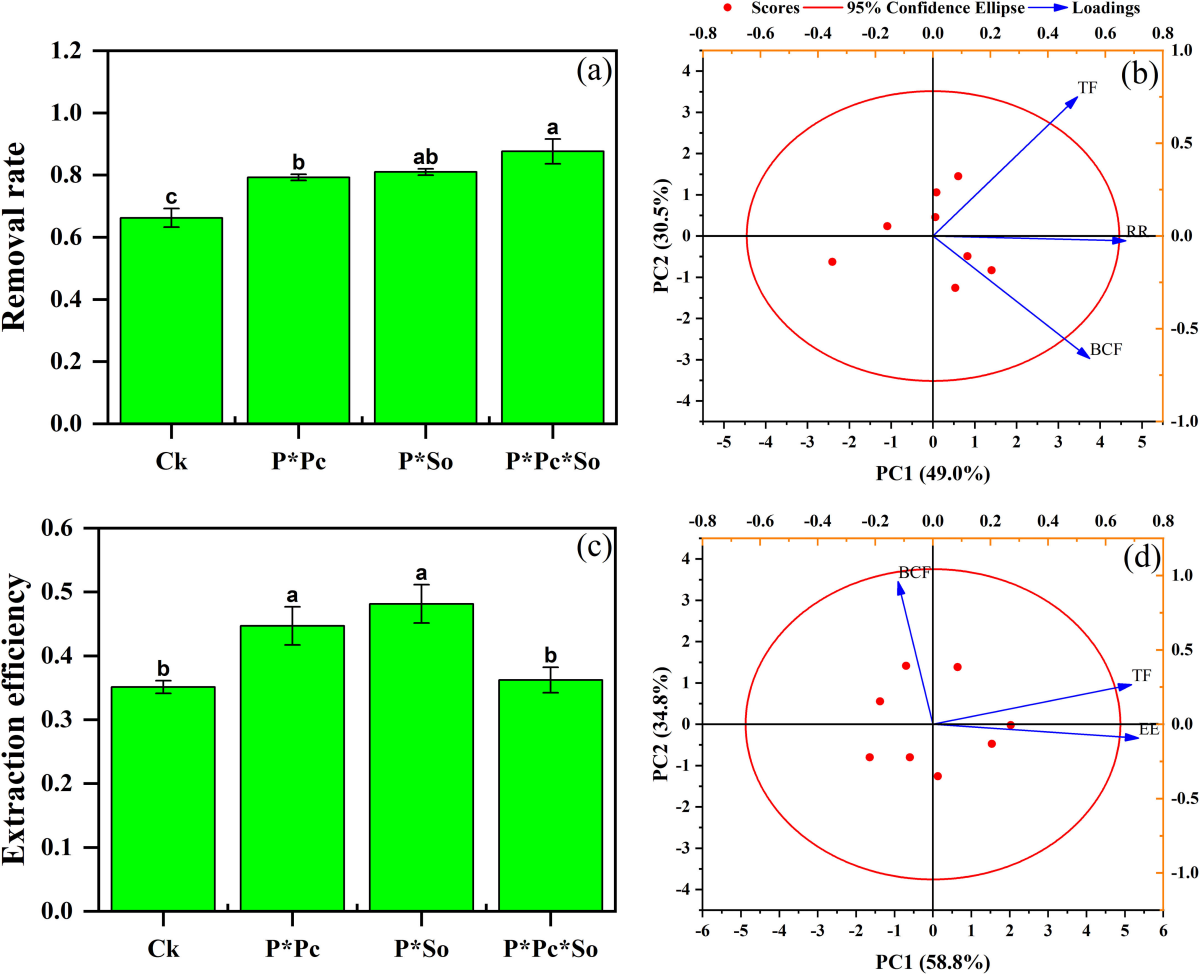
As removal rate **(a)** and PCA between As removal rate **(b)**, extraction efficiency **(c)**, and As BCF and TF under different planting patterns **(d)**. Data are presented as means ± standard errors (n = 5). Lowercase letters in the same column indicate significant treatment differences (p < 0.05). Ck refers to monocropped peanut, P ∗ Pc refers to intercropped peanut and *Pteris cretica*, peanut grown with (P ∗ So) refers to intercropped peanut and *Spinacia oleracea* L., and P ∗ Pc ∗ So refers to intercropped peanut, *Pteris cretica*, and *Spinacia oleracea* L.

The results showed a higher removal rate in the intercropping system than in the monocropping system. A significant increase in As removal rate was noted under hyperaccumulator plant diversity, with an 88% removal rate, followed by intercropped peanut/*S. oleracea* L. and peanut/*P. cretica* at 81% and 80%, respectively (**Figure 5a**). The presence and diversity of hyperaccumulators enhanced the As removal rate. In addition, principal component analysis (PCA) with a cumulative contribution ratio of 79.5% between the As removal rate and TF and/or BCF showed a positive correlation between As removal rate and TF compared to As BCF, where a negative correlation was observed (**Figure 5b**).

In contrast to the removal rate, the extraction efficiency of peanut/*P. cretica* and peanut/*S. oleracea* L. intercropping system was higher compared to under monocropped peanut and peanut/*P. cretica*/*S. oleracea* L. intercropping system. The highest extraction efficiency was observed for peanut/*S. oleracea* L. intercropping system with 29%, followed by peanut/*P. cretica* with 27%. The lowest extraction efficiency was recorded for the monocropped peanuts (21%) (**Figure 5c**). In addition, the extraction efficiency was positively correlated with the TF (**Figure 5d**).

### 3.6 Optimal planting pattern

The optimal planting pattern was defined here as higher plant height, increased removal rate, and reduced As uptake in peanut plants, as shown in **Tables 2**, **3**. The average of five replications of the optimization method based on Euclidean distances, normalized matrix, performance score, and TOPSIS ranking was used to determine the optimal peanut planting pattern. Considering peanut plant height and As uptake, the best treatment was recorded in the intercropping system with *P. cretica* (P ∗ Pc), with a performance rate of 0.886 (**Table 2**). The worst performance, considering plant height and As uptake, was recorded under P ∗ Pc ∗ So, with a performance score of 0.115, as ranked 4th (**Table 2**).

**Table 2:** TOPSIS scores and ranks of experimental treatments balancing peanut plant height and As uptake.

Treatments	Normalized matrix		Euclidean distances		Performance	
	Plant height (w=0.5)	Peanut As uptake (w=0.5)	d+	d-	Pi	TOPSIS ranking
Ck	0.265	0.194	0.165	0.074	0.310	3
P*Pc	0.235	0.356	0.043	0.238	0.886	1
P*So	0.263	0.268	0.092	0.148	0.616	2
P*Pc*So	0.235	0.120	0.236	0.031	0.115	4

w = Weight of the evaluation objective; Ck: refers to monocropped peanut, P*Pc refers to intercropped peanut and *Pteris cretica*, peanut grown with (P*So) refers to intercropped peanut and *Spinacia oleracea* L., and P*Pc*So refers to intercropped peanut, *Pteris cretica*, and *Spinacia oleracea* L.

**Table 3:** TOPSIS score and rank of each experimental treatment balancing As removal rate and As uptake by peanut plant.

Treatments	Normalized matrix		Euclidean distances		Performance	
	Removal rate (w=0.5)	Peanut As uptake (w=0.5)	d+	d-	Pi	TOPSIS ranking
Ck	0.252	0.175	0.152	0.026	0.245	4
P*Pc	0.278	0.239	0.107	0.064	0.374	3
P*So	0.256	0.242	0.091	0.070	0.435	2
P*Pc*So	0.210	0.322	0.023	0.161	0.701	1

w = Weight of the evaluation objective; Ck: refers to monocropped peanut, P*Pc refers to intercropped peanut and *Pteris cretica*, peanut grown with (P*So) refers to intercropped peanut and *Spinacia oleracea* L., and P*Pc*So refers to intercropped peanut, *Pteris cretica*, and *Spinacia oleracea* L.

Considering the As removal rate and uptake by peanut plants, the best performance was recorded in the intercropping system with hyperaccumulator plant diversity (P ∗ Pc ∗ So) with a performance score of 0.701 (ranked 1st) followed by peanut/*S. oleracea* (P ∗ So). The worst performance was observed in monocropped peanuts with a performance score of 0.245 (**Table 3**). Overall, this study demonstrated that hyperaccumulator plant diversity enhances As removal from the soil, while reducing As uptake in peanut plants.

### 3.7 Distribution of As fractions in soil

**Figure 6:**
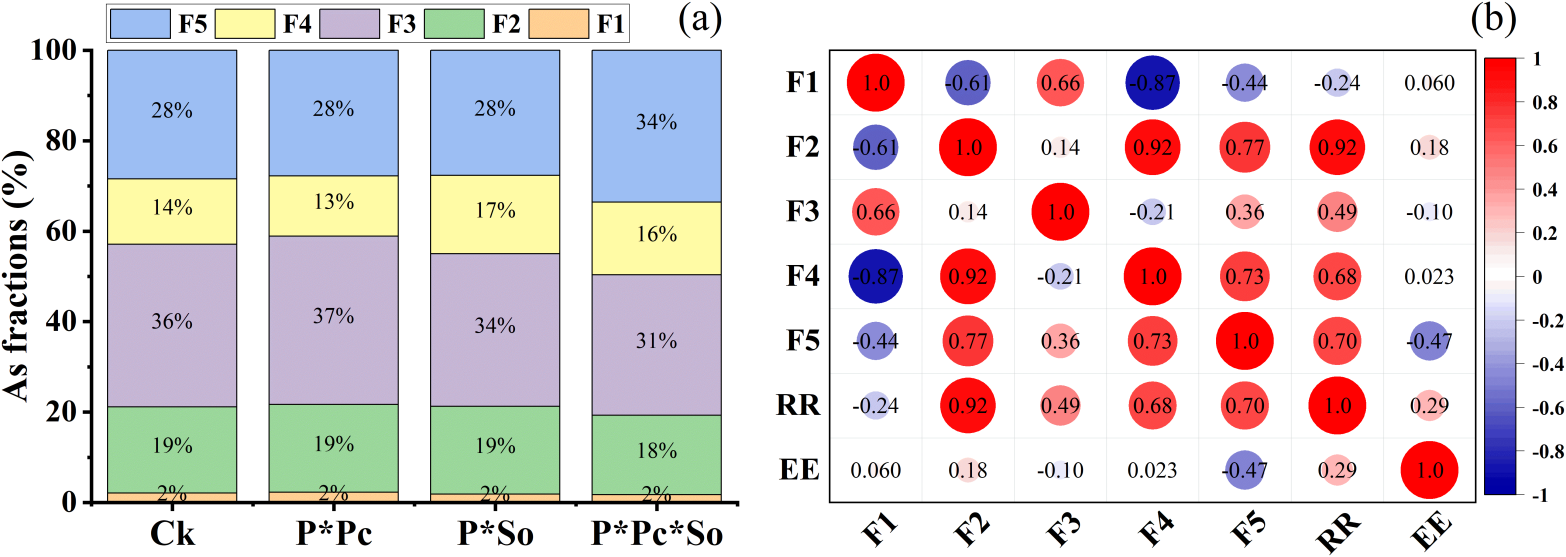
As species fractions in the rhizosphere soil under different planting patterns **(a)** and correlation between As fractions, removal rate, and extraction efficiency **(b)**. F1–F5 means different As species in rhizosphere soil. Ck refers to monocropped peanut, P ∗ Pc refers to intercropped peanut and *Pteris cretica*, peanut grown with (P ∗ So) refers to intercropped peanut and *Spinacia oleracea* L., and P ∗ Pc ∗ So refers to intercropped peanut, *Pteris cretica*, and *Spinacia oleracea* L.

The distribution of As fractions in the rhizosphere soil and the correlation between As fractions and RR/EE are shown in **Figure 6**. The results show that F1 (non-specifically sorbed) and F2 (specifically sorbed) were constant across all treatments at 2% and 19%, respectively, except for F2 under P ∗ Pc ∗ So, which showed a slight decrease to 18%. A decrease in F3 (amorphous and poorly crystalline hydrous oxides of Fe and Al) was observed under the P ∗ Pc ∗ So treatment compared to other treatments (**Figure 6a**). A predominance of F3 and F5 (residual phases) was observed across all treatments compared to F1, F2, and F4 (well-crystallized hydrous oxides of Fe and Al). In addition, F5 was higher under P ∗ Pc ∗ So at 34% and constant at 28% in the other treatments. The percentage of F1 was not significant in any of the treatments compared to the other As species. Moreover, negative correlations were observed between F1 and RR (r = −0.71) and between F1 and EE (r = −0.88) (**Figure 6b**), whereas positive correlations were observed between F3 and both RR and EE. These findings suggest that plant–soil interactions, along with hyperaccumulator diversity, significantly affect As fraction distribution in the soil.

## 4 Discussion

This study suggests that peanut growth traits were slightly decreased in the presence of hyperaccumulator plants. As previously indicated, the integration of hyperaccumulator plants into intercropping systems presents both opportunities and challenges for crop growth and soil management (Reeves et al., 2018; Hu et al., 2019). For instance, hyperaccumulator plants offer potential benefits in terms of HM-contaminated soil remediation and provide ecosystem services regarding soil health improvement and plant diversity (Peer et al., 2005; Rascio and Navari-Izzo, 2011). However, the interaction between hyperaccumulator plants and consumable crops in an intercropping system requires careful consideration to optimize agronomic outcomes and minimize potential risks. The decrease in peanut growth parameters might be attributed to the type of hyperaccumulator plants. For instance, *P. cretica* could be aggressive toward some plants and negatively affect their growth (Fayiga and Saha, 2016; Wang et al., 2017). Thus, careful selection should be made to optimize the positive effects of hyperaccumulator plants in accumulating HMs without compromising the growth of neighboring plants.

It is well known that hyperaccumulator plants possess a great ability to accumulate high concentrations of HMs from contaminated soil without significantly affecting their growth. In line with previous studies, the results suggest that hyperaccumulator plants effectively reduce As concentration in soil, with enhanced effects observed under hyperaccumulator plants’ density and diversity (Srivastava, 2017; Wang et al., 2023; Fadzil et al., 2024). It has been stated that intercropping hyperaccumulator plants with other crops can reduce health risks associated with consuming crops grown in soil contaminated with heavy metals (Hu et al., 2019; Kama et al., 2023c). Moreover, previous studies suggested that the use of hyperaccumulator plants in phytoremediation is one the most efficient methods in terms of ecological restoration of HM-contaminated soil, given its cost-effectiveness and limited landscape disturbance (Bhat et al., 2022), which could be explained by the lower concentration of As in soil observed under hyperaccumulator diversity. This study demonstrates that the diversity of hyperaccumulators improves As removal in soil without significantly hindering peanut growth.

Exploring the effects of hyperaccumulator plants on the removal rates (RR) and extraction efficiency (EE) of HMs under an intercropping system involves many aspects, including the type and diversity of hyperaccumulator plants, the status of the contaminated soil, and planting patterns. Hyperaccumulator plants absorb high concentrations of HMs from the soil and efficiently translocate and accumulate them in their aerial parts (Sharma et al., 2022; Fadzil et al., 2024). This situation explains the reduced As concentration in peanut plants grown in intercropping systems with *P. cretica* and *S. oleracea* L. decreasing the chances of peanut fruits absorbing and accumulating toxic HMs and contaminating the food chain. The reduced concentration of As in peanuts grown under an intercropping system with *P. cretica* and *S. oleracea* L. represents a significant advancement in lowering heavy metal levels in edible crops with underground fruits. This study demonstrates that the peanut/*P. cretica*/*S. oleracea* L. intercropping system reduces the potential risk of HM food contamination through the underground fruit of crops cultivated in contaminated soil. In addition, it is noted that the concentration of As in the consumable parts of the spinach plant was within the permissible limits for consumption, and its choice could be explained by the high accumulation capacity of HMs in its roots (Pavlík et al., 2010; Veladzic et al., 2015).

This study provided evidence that hyperaccumulator plants significantly reduced As accumulation in peanut plants while decreasing As concentration in the soil. These findings are consistent with previous studies indicating that the presence and diversity of hyperaccumulators can reduce the potential health risks associated with As accumulation in peanut fruits (Peng et al., 2021). In addition, it was also found that the residual phase (F5) of As species was higher under hyperaccumulator diversity compared to other treatments, demonstrating the significant effects of hyperaccumulator plants in soil As degradation (Vandana et al., 2020). Intercropping peanuts with hyperaccumulator plants capable of absorbing and sequestering high concentrations of As from the soil is a promising strategy to mitigate potential As accumulation in peanut fruits. It is well known that As fractions have different mobility, bioavailability, and toxicity toward plants (Nabi et al., 2021). For instance, it was found that the hyperaccumulator plant *Pteris vittata* (Chinese brake fern) can absorb and store large amounts of As in its tissues without suffering toxicity (Cui et al., 2023). The results support previous studies suggesting that intercropping systems reduce As bioavailability through the uptake of soluble and exchangeable As (Bhattacharya et al., 2021; Kumar et al., 2022). Hyperaccumulators primarily target the most mobile fractions, particularly the water-soluble and exchangeable forms of As (Bhattacharya et al., 2021; Cui et al., 2023). The absorption of As by hyperaccumulator plants from these fractions could reduce the pool of As available for uptake by intercropped crops such as peanuts. This study demonstrates that intercropping peanuts with hyperaccumulator plants offers a viable strategy for reducing As contamination in peanut crops by altering the distribution of As fractions in the soil. Hyperaccumulators effectively reduce the bioavailable As fractions, lowering the risk of uptake by peanuts. These positive impacts of hyperaccumulator plants in the phytoremediation process, without causing adverse effects on the cultivated land and the target crops, explain the considerable public interest in hyperaccumulator plants.

## 5 Conclusion

This study demonstrates the promising potential of hyperaccumulator diversity and intercropping systems in simultaneously decreasing soil As content and reducing As uptake in peanut plants. Intercropping diverse hyperaccumulator species significantly improved As removal efficiency over monocropping. Moreover, the presence of hyperaccumulator species has significantly reduced peanut As uptake, minimizing the risk of As transfer to the food chain. These findings highlight the significance of integrating biodiversity with innovative agricultural practices to tackle soil contamination issues and ensure food security. The concentration of As content in the spinach plant shoot, which is a consumable crop, was under permissible limits, reducing the potential risk of affecting the food chain. However, further investigations are necessary to optimize the underlying mechanisms and evaluate long-term efficacy and sustainability. In addition, investigating and assessing the effects of hyperaccumulator plant diversity and density on HMs removal under intercropping systems with peanuts in natural fields, as well as assessing the risk to underground fruits of peanuts, would be a significant step toward safe peanut production in HM-contaminated soils.

## Data Availability

The raw data supporting the conclusions of this article will be made available by the authors, without undue reservation.
